# First-Principle Study of the Optical Properties of Dilute-P GaN_1−x_P_x_ Alloys

**DOI:** 10.1038/s41598-018-24384-1

**Published:** 2018-04-16

**Authors:** Damir Borovac, Chee-Keong Tan, Nelson Tansu

**Affiliations:** 1 Center for Photonics and Nanoelectronics, Department of Electrical and Computer Engineering, Lehigh University, Bethlehem, PA 18015 USA; 2 Department of Electrical and Computer Engineering, Clarkson University, Potsdam, NY 13699 USA

## Abstract

An investigation on the optical properties of dilute-P GaN_1−x_P_x_ alloys by First-Principle Density Functional Theory (DFT) methods is presented, for phosphorus (P) content varying from 0% up to 12.5%. Findings on the imaginary and real part of the dielectric function are analyzed and the results are compared with previously reported theoretical works on GaN. The complex refractive index, normal-incidence reflectivity and birefringence are presented and a difference in the refractive index in the visible regime between GaN and GaNP alloys of ~0.3 can be engineered by adding minute amounts of phosphorus, indicating strong potential for refractive index tunability. The optical properties of the GaN_1−x_P_x_ alloys indicate their strong potential for implementation in various III-nitride-based photonic waveguide applications and Distributed Bragg Reflectors (DBR).

## Introduction

The soaring interest for the advancement of III-nitride semiconductors has been primarily driven by their highly desirable electronic, optoelectronic, chemical and tribological properties, suitable for a wide range of applications^1–8^. In recent years, the binary (Al, Ga, In) N alloys and their respective ternary and quaternary alloys, have been in the epicenter of the solid-state lighting revolution, led by the development of innovative material epitaxy techniques^9–14^. The impact produced by advances in III-nitride semiconductors was recognized by the 2014 Nobel Prize in Physics, awarded for the inventions of violet and blue GaN-based light emitting diode (LED) technologies^15^.

Despite the successful integration of InGaN-based active regions targeting the emission for blue and green spectral regimes, the III-nitride field still faces two major challenges. Firstly, III-nitride-based LEDs suffer from the efficiency-droop issue that occurs at high operating current densities, attributed to Auger recombination and carrier leakage^16–20^. Secondly, the quest to push for longer wavelength emission beyond the green spectral regime by using In_x_Ga_1−x_ N-based devices has been stagnating, mainly attributed to material quality issues at high indium contents (x > 0.2), resulting in low external quantum efficiencies (EQE) ~ 4% of the red-emitting LEDs^21–25^. Several methods have been proposed to overcome the two issues, which includes innovative material systems and active region design suitable for emission in the longer wavelength regimes^26–32^. Recently, dilute-anion III-nitride semiconductors such as dilute-As GaNAs and dilute-P GaNP have been suggested as alternative candidates to InGaN, due to their highly desirable electronic properties, including the possibility to suppress the Auger recombination rate and capability to push for longer wavelength emission^31–33^. Moreover, a novel active region design utilizing the InGaN/GaNAs interface quantum well (IQW) concept has been proposed by Tan and co-workers, with the possibility of enhancing the spontaneous emission rate by ~8.5 times compared to that of the conventional InGaN QW^34^. However, the development of dilute-anion III-nitride-based semiconductors is still in the early stage, specifically dilute-P GaNP alloys^35–44^, when compared to the common-anion dilute-nitride (In)GaAs(P)-based semiconductors, which are well-established material systems in the telecommunication applications^45–48^.

The incorporation of phosphorus (P) impurities into GaN was first successfully performed using the halide vapor phase epitaxy (HVPE) technique by Igarashi^35^. Then, using the molecular beam epitaxy (MBE) technique, Iwata and co-workers synthesized the dilute-P GaNP material and have shown a red-shift of ~0.15 eV by incorporating minute amounts of phosphorus (~1–2%) into the GaN-based system^36^. Moreover, Yoshida and co-workers successfully integrated the dilute-P GaNP material into a single quantum well (SQW) light emitting device by using the metal organic chemical vapor deposition (MOCVD) technique^37^. However, despite the previously demonstrated epitaxial feasibility of the dilute-P GaNP semiconductor, there remains a lack of knowledge about the fundamental material properties instrumental for grasping the full potential of this III-nitride-based material system.

The promising electronic properties of the dilute-P GaNP semiconductor and its experimental feasibility pose it as an excellent candidate for applications for visible light emission. Nevertheless, recognizing their full potential requires detailed studies beyond the electronic properties. In particular, the optical properties of the dilute-P GaNP will play a significant role in the understanding of the behavior of the dilute-P GaNP alloys and their role in future photonic device application, including those of distributed Bragg reflectors (DBR) and photonic crystals^49–51^. Therefore, it is of great importance to understand the underlying optical properties of the dilute-P GaNP alloys and their implication for future design of III-nitride-based photonic devices.

In this work, First-Principle Density Functional Theory (DFT) calculations with an applied scissor operator were performed to investigate the optical properties of dilute-P GaN_1−x_P_x_ alloys, with phosphorus (P) content ranging from 0% up to 12.5%. The imaginary part of the complex dielectric function is initially calculated and Kramers-Kronig (KK) transformation was performed to obtain the real part of the complex dielectric function. Optical constants including the complex refractive index, normal-incidence reflectivity and birefringence are derived based on the complex dielectric function and comparisons are drawn to other III-nitride material systems. This work elucidates on the optical properties of the dilute-P GaNP alloys and the capability to tune the refractive index by inserting minute amounts of phosphorus into the GaN-based material, of which are essential for enabling the device-level incorporation of these materials.

### Computational Method

**Figure 1:**
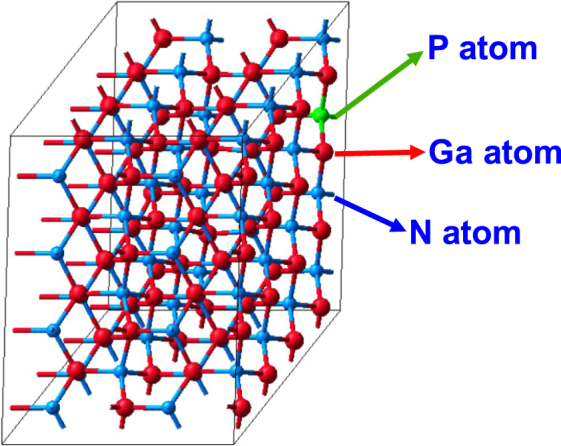
A 128-atom dilute-P GaNP supercell, with 1 phosphorus atom replacing a nitrogen atom, corresponding to 1.56% P-content.

For building the appropriate crystal structures of the dilute-P GaNP alloys required for calculating the optical properties, our DFT calculations employ the supercell approach. Figure 1 illustrates a 128-atom supercell consisting of 64 gallium (Ga) atoms, 63 nitrogen (N) atoms and 1 phosphorus (P) atom, resulting in 1.56% P-content in the GaN_1−x_P_x_ alloy. The phosphorus content in the dilute-P GaNP alloys is varied by varying the supercell size and by replacing one nitrogen atom with a phosphorus atom at a time, which is a common approach in supercell calculations^52^. The calculations utilize the projector augmented wave (PAW) method within the Vienna ab initio simulation package (VASP)^53,54^. Additionally, for structure optimization, the Generalized Gradient Approximation (GGA) with the exchange-correlation potentials by Perdew, Burke and Ernzerhof (PBE) was employed^55^. The atoms were allowed to relax with a Hellman-Feynman force of 0.02 eV/Å, while the energy convergence criterion was set to 1 × 10^−5^ eV/atom. Thus, the optical properties were then calculated using the Local Density Approximation (LDA) and by applying the appropriate scissor operator to shift the imaginary part of the dielectric function with respect to the photon energy, similar to previous works^56^. The scissor operator is inversely proportional to the dielectric constant and was therefore varied based on the phosphorus content in the dilute-P GaNP alloys. Note that the method for calculating the optical properties is similar to the one reported previously for the case of dilute-As GaNAs alloys^57^. The plane-wave cutoff energy was set to 400 eV, while the external stress applied to the system was set to 0 GPa. For obtaining the optical properties, the tetrahedron smearing method was chosen as the integration scheme, and our calculations were based on the blocked Davidson algorithm. Moreover, the k-meshes sizes and the number of bands varied depending on the supercell size, where the 128-atom supercell had a 3 × 3 × 3 mesh, and the 16-atom supercell a 9 × 9 × 9 mesh. Thus, our calculations exclude spin-orbit coupling since we determined that the effect of phosphorus was negligible, similar to other wide band-gap III-nitride semiconductors.

### Complex Dielectric Function for Dilute-P GaNP Alloys

For the calculation of the optical properties of the dilute-P GaNP alloys, the complex dielectric function as a function of energy E is defined as $\varepsilon (E)={\varepsilon }_{1}(E)+i{\varepsilon }_{2}(E)$, where ${\varepsilon }_{1}(E)$ and ${\varepsilon }_{2}(E)$ are the real and imaginary parts of the complex dielectric function, respectively. By using the Kramers-Kronig relation, the real and imaginary parts of the complex dielectric function are described as follows^58^:

1 $${\varepsilon }_{1}(E)=1+\frac{2}{\pi }{\int }_{0}^{\infty }\frac{E^{\prime} {\varepsilon }_{2}(E^{\prime} )}{{E^{\prime} }^{2}-{E}^{2}}dE^{\prime} ,$$

2 $${\varepsilon }_{2}(E)=\frac{-2E}{\pi }{\int }_{0}^{\infty }\frac{{\varepsilon }_{1}(E^{\prime} )}{{E^{\prime} }^{2}-{E}^{2}}dE^{\prime} .$$

Thus, when the information on the imaginary part of the dielectric function is known, the real part of the dielectric function can be calculated using equation (1).

**Figure 2:**
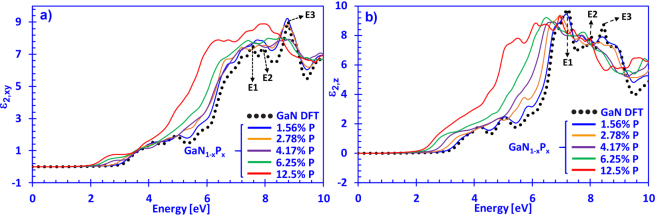
(**a**) Imaginary part of the dielectric function for **E ⊥ c** direction (*ε*_2_,_*xy*_(*E*)) and (**b**) **E** ‖ **c** direction (*ε*_2_,*Z*(*E*)) spectra for dilute-P GaNP alloys, with P-content varying from 0% up to 12.5% phosphorus, respectively.

In Fig. 2(a,b) the imaginary parts of the complex dielectric function of dilute-P GaN_1−x_P_x_ alloys with phosphorus content varying from 0% up to 12.5% in the xy- and z-directions are shown, respectively. The xy-direction is defined as the direction where the polarization vector is perpendicular to the surface of the GaNP alloys (**E** ⊥ **c**), while the z-direction is the case where the polarization vector is parallel (**E** ‖ **c**) to the surface (c-axis) of the GaNP alloys. The two polarization directions arise from the anisotropic behavior of wurtzite material systems, where examples include other III-nitride material systems like (Al, Ga, In)N and their respective alloys^59,60^.

Figure 2(a) indicates that increasing the P-content in the dilute-P GaNP alloys shifts the imaginary dielectric function spectra in the xy-direction towards lower photon energies. The phenomenon shown in Fig. 2(a) is directly related to the decrease in energy band gap when P-content increases in dilute-P GaNP alloys, which has been observed experimentally and theoretically^32,36^. Figure 2(a) also shows the E1, E2 and E3 absorption peaks for GaN occurring at around 7.8 eV, 8.3 eV and 9.1 eV, respectively, aligning well with previously reported values in which E1, E2 and E3 peaks are positioned around 7.5 eV, 7.9 eV and 9 eV, respectively^61,62^. Interestingly, the curvature of the ${\varepsilon }_{2,xy}(E)$ spectra for dilute-P GaNP alloys in the vicinity of the E1–E3 peaks is broader compared to that of DFT-calculated GaN, possibly stemming from the formation of localized P-impurity states and their influence on the optical transitions within the Brillouin Zone (BZ). Thus, increasing the P-content in the GaN-based alloy perturbed the shape and magnitude of the major absorption peaks for all of the P-contents calculated, with the 12.5% P-containing alloy exhibiting the strongest modification of the ${\varepsilon }_{2,xy}(E)$ spectra.

From Fig. 2(b), it can be observed that the E1, E2 and E3 absorption peaks have their respective positions located around 6.9 eV, 8.1 eV and 8.9 eV for GaN in the z-direction for the ${\varepsilon }_{2,z}(E)$ spectra. Our results on the imaginary parts of the dielectric function of GaN also show strong agreement with previous results for the locations of the E1–E3 peaks for the GaN alloy, indicating the validity of our calculation methods. Moreover, increasing the phosphorus content shifts the ${\varepsilon }_{2,z}(E)$ spectra towards lower photon energies, which is similar to the case of the xy-direction. Figure 2(b) indicates that the onset of the imaginary part of the dielectric function for the 12.5% P-containing alloy is around ~2.7 eV, which is in good agreement with the energy band gap reported by Tan and co-workers^32^. Additionally, note that the E1–E3 group of absorption peaks has been reported to correspond mostly to optical transitions near *M*, but contributions from *U*, *S*’, *Σ, H* and *K* points in the BZ are present^63^. For the sake of the dilute-P GaNP alloys, further studies are required to elucidate on the origin of the optical transitions due to the influence of P-impurities in GaN. The ${\varepsilon }_{2}(E)$ spectra for both, xy- and z-directions exhibits significant perturbations when the P-atoms are incorporated and is similar to the behavior of the ${\varepsilon }_{2,z}(E)$ spectra reported the case of dilute-As GaNAs alloys^57^. Also, it should be noted that this work does not account for several effects such as strain, temperature and alloy disorder, which have previously been reported to affect optoelectronic properties of III-nitride semiconductors, especially in the vicinity of the energy band gap^1^. Thus, the optical properties reported here can serve as a guide for experimentalists pursuing this material in the future and can be useful in future simulations focusing on photonic waveguide and optical modulation applications.

**Figure 3:**
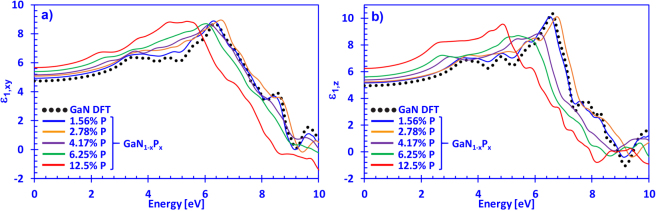
(**a**) Real part of the dielectric function for **E ⊥ c** direction (*ε*_1_,_*xy*_(*E*)) and (**b**) **E** ‖ **c** direction (*ε*_1_,*Z*(*E*)) spectra for dilute-P GaNP alloys, with P-content varying from 0% up to 12.5% phosphorus, respectively.

In Fig. 3(a,b) the calculated real part of the dielectric spectra of dilute-P GaN_1−x_P_x_ alloys is shown, with P-composition reaching from 0% up to 12.5%, for the xy- and z-directions, respectively. The data was obtained by performing the Kramers-Kronig transformations on the imaginary part of the dielectric function by using the above-mentioned equation (1). The calculated data for the ${\varepsilon }_{1,xy}(E)$ spectra for GaN agrees well in magnitude and overall shape of the ${\varepsilon }_{1,xy}(E)$ spectra with the theoretical and experimental results obtained by Benedict and co-workers^64^. Moreover, Fig. 3(a) shows that the real part of the dielectric function is an increasing function of the phosphorus content in the dilute-P GaNP alloy, with the 12.5% P-containing alloy having the largest magnitude for energies up to ~5 eV. Thus, the high-frequency dielectric constant can be seen from Fig. 3(a), where the value of ${\varepsilon }_{xy,\infty }$ increases for increasing P-content, where the GaN_0.875_P_0.125_ alloy has a value of ${\varepsilon }_{xy}(\infty )\, \sim \,\,$5.65. Additionally, the high-frequency dielectric constant for GaN in this work is in excellent agreement with previous works by Lambrecht *et al*. who obtained ${\varepsilon }_{xy}(\infty )$ ~ 4.79, while we obtained a value of ${\varepsilon }_{xy}(\infty )$ ~ 4.8^65^. By the high-frequency dielectric constant, we refer to the dielectric constant at frequencies much lower than any electronic transitions and much higher than any typical phonon frequency. Hence, this work elucidates on the basic understanding of some of the optical properties of the dilute-P GaNP alloys, the information obtained here can be useful in device simulation and modeling aimed at photonic applications using the dilute-P GaNP material.

Figure 3(b) indicates that our DFT-calculated GaN results for the ${\varepsilon }_{1,z}(E)$ spectra are in good agreement with those obtained by Benedict and co-workers^64^. Moreover, the high-frequency dielectric constant ${\varepsilon }_{z}(\infty )$ for GaN obtained was ~ 4.95, which is lower compared to that of Carvalho *et al*. (~5.3) and higher than that of Christensen *et al*. (~4.62)^63,66^. It should be noted that the slight discrepancy between this work and those of Christensen and co-workers is due to the different treatment of the LDA-based calculation, since they did not apply any correction terms like the scissor operator to adjust the calculation for possible errors. Thus, increasing the phosphorus content in the dilute-P GaNP alloys lead to a linear increase of the high-frequency dielectric constant, with the 12.5% P-containing alloy having the largest value of ${\varepsilon }_{z}(\infty )$ ~ 6.2 compared to all other P-contents considered. Additionally, the z-direction real part of the dielectric function is slightly larger in magnitude compared to the xy-direction for energies up to ~5 eV, and can be attributed to the anisotropic behavior of the wurtzite crystal structure. This is similar to other III-nitride systems, which has been experimentally observed by Pezzagna and co-workers^67^.

### Complex Refractive Index, Reflectivity and Birefringence of Dilute-P GaNP Alloys

From the information on the complex dielectric function, other optical constants including the complex refractive index and normal incidence reflectivity can be obtained. The optical constants are governed by the following formulas^58^:

3 $$n(E)=\sqrt{\frac{\sqrt{{\varepsilon }_{1}{(E)}^{2}+{\varepsilon }_{2}{(E)}^{2}}+{\varepsilon }_{1}{(E)}^{2}}{2}},$$

4 $$k(E)=\sqrt{\frac{\sqrt{{\varepsilon }_{1}{(E)}^{2}+{\varepsilon }_{2}{(E)}^{2}}-{\varepsilon }_{1}{(E)}^{2}}{2}}.$$

**Figure 4:**
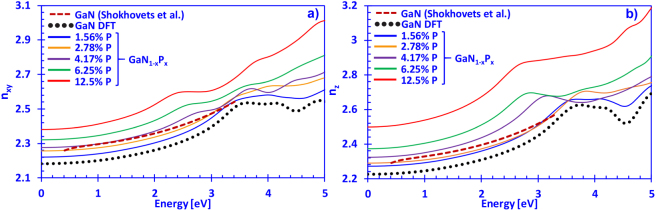
(**a**) Refractive index in the xy-direction (*η*_*xy*_(*E*)) and (**b**) z-direction (*η*_*z*_(*E*)) spectra for dilute-P GaNP alloys (solid lines), with P-content ranging from 0% up to 12.5% phosphorus, and experimentally fitted data (dashed lines), respectively.

Here, the complex refractive index is defined as ${n}^{\ast }(E)=n(E)+ik(E)$, where $n(E)$ is the real part and $k(E)$ is the imaginary part of the complex refractive index, respectively. Figure 4(a,b) illustrate the xy- (${n}_{xy}(E)$) and z-directions (${n}_{z}(E)$) of the real part of the refractive index for dilute-P GaNP alloys with P-content ranging from 0% up to 12.5%, respectively. For comparison purposes, the experimentally fitted data for the refractive index of GaN by Shokhovets and co-workers has been plotted^68^. From Fig. 4(a), increasing the phosphorus content in the dilute-P GaNP alloys is linearly proportional to the increase in the refractive index of the GaNP material for photon energies within the visible regime. The refractive index difference between GaN and GaNP can be potentially engineered to be about ~0.3 with 12.5% P-content in GaNP, and possibly even higher with P-content above 12.5%. However, in this study we have limited the phosphorus content to 12.5% and the effect of higher P-content will require further detailed studies. It should be noted that the Fig. 4(a,b) do not indicate the regions where the dilute-P GaNP alloys will start absorbing (band edges). Additionally, the experimentally-fitted data from Shokhovets and co-workers agreed well with our DFT-calculated results for GaN, with only about ~5% difference on average (up to the band edge). The discrepancy can be attributed to several effects such as surface roughness and strain not being considered in our DFT-calculations, which have been previously reported to have a strong influence on the optical properties of GaN^69^. This information will prove instrumental for photonic device design based on this material system, but will be essential for future studies.

From Fig. 4(b), the refractive index in the z-direction for the DFT-calculated GaN is also in strong agreement with the data reported by Shokhovets and co-workers, with an average difference of about ~5%, similar to the xy-direction in Fig. 4(a). It should also be noted that Rigler and co-workers have reported that the polarity of the III-nitride system, depending on whether it is Ga-polar or N-polar, can create a variation in the refractive index, which has been ascribed to the varying impurity contents and has not been considered in this study^70^. Similar to the xy-direction, the increase of the refractive index in the z-direction as compared to DFT-calculated GaN can be significant (~0.3). Moreover, for both, the xy- and z-directions, increasing the phosphorus content in the dilute-P GaNP alloys shifts the band edge peaks towards the lower photon energies which is in agreement with the energy gap reduction reported previously, where the 12.5% P-containing GaNP alloy has a band edge peak around ~ 2.7 eV^32^. In other conventional III-nitride material systems like AlGaN, the refractive index is also a linear function of the Al-content, as is for the case of the dilute-As GaNAs alloys^57,71^. However, in the InGaN material system, the refractive index is much more difficult to control and predict, since it is heavily dependent on intrinsic properties like the piezoelectric-field induced Stark-effect as well as variations due to quantum-confinement^22,72^. Therefore, by precisely controlling the P-content in the dilute-P GaNP alloys, it is possible to tune the refractive index of the GaN-based system and thereby achieve high index difference compared to GaN.

**Figure 5:**
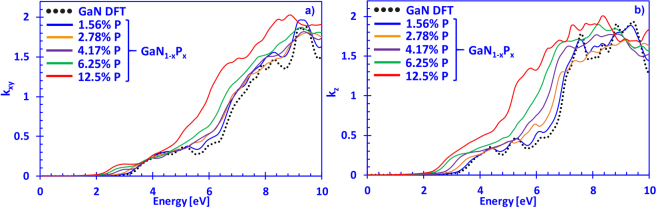
(**a**) Imaginary part of the complex refractive index in the xy- (*E*) and (**b**) z-direction (*E*) spectra for dilute-P GaNP alloys, with P-content ranging from 0% up to 12.5%, respectively.

In Fig. 5(a,b), the imaginary parts of the refractive index in the xy- and z-directions for dilute-P GaNP alloys, with phosphorus content varying from 0% up to 12.5% are shown, respectively. The Fig. 5(a,b) indicate that the increase in the phosphorus content perturbs the k_xy_ and k_z_ spectra significantly and shifts the onsets of absorption to lower photon energies, which is also related to the changes in the band structure of the dilute-P GaNP material system^32^. It has been shown previously that the P-impurity level lies above the top-most valence band of GaN (~0.2 eV), and that the introduction of P atoms leads to an upward movement of the top-most valence band due to the formation of localized P states^43,73^. This behavior is similar to the phenomenon observed in dilute-As GaNAs alloys, where the As impurities lie about ~0.4 eV above the top-most valence band of GaN and form localized As states^73^. Therefore, due to the influence of the P-impurities, the optical spectra of the dilute-P GaNP alloys is largely modified compared to the GaN system. In comparison to other works on GaN, the shape of the DFT-calculated ${k}_{xy}(E)$ and ${k}_{z}(E)$ spectra in this work is in good agreement with those reported by Lambrecht and co-workers, while the overall magnitude is slightly lower compared to findings of Takeuchi and Djurišić with co-workers^65,74,75^. The discrepancy between findings is attributed to different treatments of the calculations.

The reflectivity at normal-incidence can also be calculated and is related to the complex refractive index in the following way:

5 $$R(E)=\frac{{[n(E)-1]}^{2}+k{(E)}^{2}}{{[n(E)+1]}^{2}+k{(E)}^{2}}.$$

**Figure 6:**
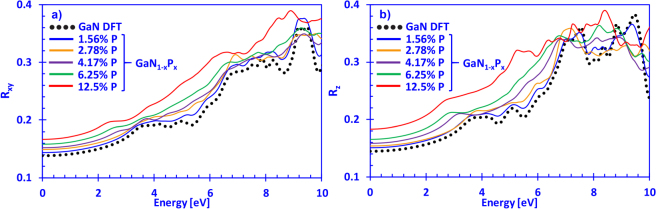
(**a**) Reflectivity at normal-incidence in the xy- ((*E*)) and (**b**) z-directions ((*E*)) for dilute-P GaNP alloys, with P-content ranging from 0% up to 12.5% phosphorus, respectively.

Figure 6(a,b) show the reflectivity at normal-incidence for the xy- (${R}_{xy}(E)$) and z-directions (${R}_{z}(E)$) for dilute-P GaNP alloys with P-content ranging from 0% up to 12.5%, respectively. As the previous Fig. 4(a,b) indicated a linear increase in the refractive index of the dilute-P GaNP alloys with the increase of phosphorus content, the behavior of the normal-incidence reflectivity follows that trend, with the 12.5% P-containing GaNP alloy having a (${R}_{xy}(E)$) in the range from ~0.16–0.20 (up to the respective band edge in ref.^32^), and (${R}_{z}(E)$) from ~0.18–0.22. The DFT-calculated results for GaN in the xy-direction (Fig. 6(a)) are in good agreement with the values reported by Kawashima and co-workers, where our values for (${R}_{xy}(E)$) vary from ~0.13–0.18 for energies up to 3 eV^76^. From Fig. 6(b), however, the normal-incidence reflectivity in the z-direction for GaN is in the range of ~0.14–0.18, slightly higher than the one shown in Fig. 6(a) for the case of the xy-direction. Similar behavior has been observed in the dilute-As GaNAs alloys, where increasing the As-content leads to an increase in the normal-incidence reflectivity, particularly up to the respective band edges of the alloy compositions considered in ref.^57^.

**Figure 7:**
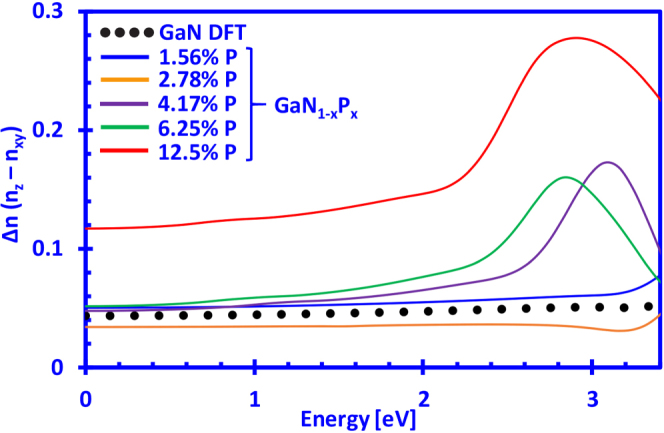
Birefringence (Δn) of dilute-P GaN_1−x_P_x_ alloys with P-content ranging from 0% up to 12.5%.

Furthermore, the information on the real part of the refractive index of dilute-P GaNP alloys can be useful for determining the birefringence of the GaN-based alloys. The birefringence is defined as the difference in the refractive index between the xy- and z-directions (n_xy_ and n_z_) and can be written as Δn = n_z_ − n_xy_. In Fig. 7 the birefringence of dilute-P GaNP alloys as a function of photon energy is presented, where the P-content is varied from 0% up to 12.5%. Previously reported values for GaN by Hui and co-workers have shown a birefringence of Δn ~ 0.04, while Pezzagna and co-workers measured a value of Δn ~ 0.0425^67,77^. Moreover, Shokhovets and co-workers have shown that the birefringence is an increasing function for photon energies up to the band gap of GaN and that it varies between 0.037–0.056^68^. Our DFT-calculated results show similar behavior for GaN and that Δn lies in the range from 0.044 up to 0.051 as it approaches the band edge. The Δn for P-contents up to 6.25% in the GaNP alloys are close to that of GaN up to photon energies of ~1 eV, while the 12.5% P-containing GaNP alloy has a much larger birefringence compared to the rest. On the other hand, it can be noted that at minute amounts of phosphorus in the GaNP alloy (~3%), the birefringence drops below the one of GaN and that it lies in the range of ~0.033–0.036. The decrease is relatively small (~0.01) and should not be taken as an absolute value, but rather as an indication of a possible trend of the GaNP alloy, which will require experimental confirmation. A similar trend has been observed in previous DFT-calculations for the case of dilute-As GaNAs alloys, where the birefringence of the 1.56% As-containing alloys drops below the one of GaN for photon energies approaching the band edge^57^. Although further investigation is required to elucidate on the temperature dependence of the refractive index, as well as the birefringence, the results in this work indicate a possibility to reduce or control the birefringence by simply inserting low amounts of phosphorus into the GaN material system.

## Conclusion

In summary, optical properties calculations of dilute-P GaNP semiconductor alloys with P-content varying from 0% to 12.5% have been performed by employing First-Principle DFT calculations with the LDA approximation. The imaginary part of the complex dielectric function was initially calculated and Kramers-Kronig transformations were performed to calculate the real part of the complex dielectric function. The information on the complex dielectric function was used to obtain other optical constants, including the complex refractive index and reflectivity at normal-incidence. The analysis indicates that the dilute-P GaNP alloys exhibit anisotropic behavior as they retain the wurtzite crystal structure like GaN, which resulted in two different polarization directions of the optical properties (xy- and z-directions). Moreover, the birefringence of the dilute-P GaNP alloys was discussed and results were compared to previous works on GaN and a potential pathway to reduce the birefringence has been observed. The refractive index of the dilute-P GaNP alloys showed a linear relationship as the phosphorus content was increased. Thus, our study shows that the refractive index difference between GaN and dilute-P GaNP alloys can be large (up to ~0.3) for a wide range of photon energies, with room for further enhancement at even higher phosphorus contents. The results indicate a possibility of tuning the refractive index by tweaking the phosphorus content in the dilute-P GaNP alloys, highly desirable for the design of waveguides and distributed Bragg reflectors that require a large range of refractive index differences compared to GaN. Our findings suggest the strong potential of dilute-P GaNP alloy for photonic device applications and provide the necessary information on the optical properties suitable for future photonic device simulations based on the dilute-P GaNP material system.
